# Evaluation of Additive Neuroprotective Effect of Combination Therapy for Parkinson’s Disease Using In Vitro Models

**DOI:** 10.3390/antiox14040396

**Published:** 2025-03-27

**Authors:** Alexander Shtilbans, Elise Esneault, Florian Simon, Joseph R. Mazzulli, Drew J. Quiriconi, Dror Rom, Wolfgang E. Reintsch, Andrea I. Krahn, Thomas M. Durcan

**Affiliations:** 1 Department of Neurology, Hospital for Special Surgery, New York, NY 10021, USA; 2 Department of Neurology, Weill Cornell Medicine, New York, NY 10021, USA; 3 Porsolt Research Laboratory, 53940 Le Genest-Saint-Isle, France; eesneault@porsolt.com (E.E.); florian.simon@nuvisan.com (F.S.); 4 Department of Neurology, Feinberg School of Medicine, Northwestern University, Chicago, IL 60611, USA; jmazzulli@northwestern.edu (J.R.M.); drew.quiriconi@northwestern.edu (D.J.Q.); 5 Prosoft Clinical, Chesterbrook, PA 19087, USA; d.rom@prosoftclinical.com; 6 The Neuro’s Early Drug Discovery Unit (EDDU), McGill University, Montreal, QC H3A 2B4, Canada; wolfgang.e.reintsch@mcgill.ca (W.E.R.); andrea.krahnroldan@mail.mcgill.ca (A.I.K.); thomas.durcan@mcgill.ca (T.M.D.)

**Keywords:** ROS, anti-inflammatory effects, sodium phenylbutyrate, exenatide, tauroursodeoxycholic acid, Parkinson’s disease, in vitro models, iPSCs, combination therapy, drug repurposing

## Abstract

Background: All the processes leading to neurodegeneration cannot be addressed with just one medication. Combinations of drugs affecting various disease mechanisms concurrently could demonstrate improved effect in slowing the course of Parkinson’s disease (PD). Objective: This was a drug-repurposing experiment designed to assess several combinations of nine drugs for possible added or synergistic efficacy using in vitro models of PD. Methods: We evaluated 44 combinations of the nine medications (sodium phenylbutyrate, terazosin, exenatide, ambroxol, deferiprone, coenzyme-Q10, creatine, dasatinib and tauroursodeoxycholic acid) selected for their previously demonstrated evidence of their impact on different targets, showing neuroprotective properties in preclinical models of PD. We utilized wild-type induced pluripotent stem-cell-derived human dopaminergic neurons treated with 1-methyl-4-phenylpyridinium for initial screening. We retested some combinations using an idiopathic PD patient-derived induced pluripotent stem cell line and alpha-synuclein triplication line. We assessed anti-neuroinflammatory effects using human microglia cells. As metrics, we evaluated neurite length, number of branch points per mm^2^, the number of live neurons, neurofilament heavy chain and pro-inflammatory cytokines. Results: We have identified four combinations of two to three drugs that showed an additive protective effect in some endpoints. Only the combination of sodium phenylbutyrate, exenatide and tauroursodeoxycholic acid showed improvement in four endpoints studied. Conclusions: We demonstrated that some of the medications, used in combination, can exert an additive neuroprotective effect in preclinical models of PD that is superior to that of each of the compounds individually. This project can lead to the development of the first treatment for PD that can slow or prevent its progression.

## 1. Introduction

**Figure 1:**
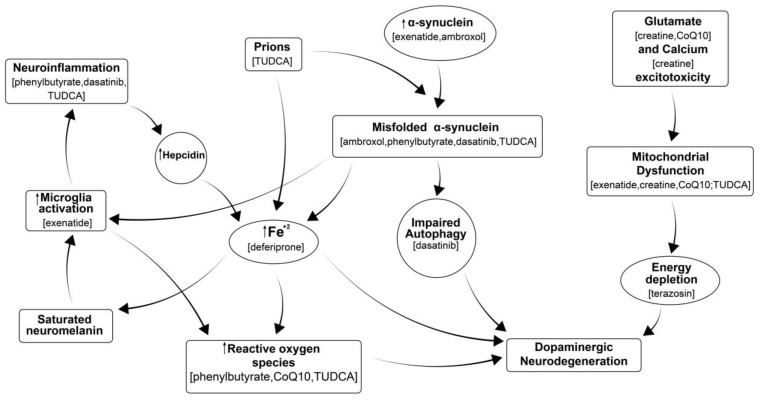
Multiple processes leading to neurodegeneration in PD include: misfolding of proteins that fail to be cleared from the brain, which in turn stimulates neuroimmune responses; calcium excitotoxicity, affecting mitochondria and resulting in energy depletion, leading to neurodegeneration; iron accumulation that leads to activation of microglia and further neuroinflammation, causing oxidative stress, formation of reactive oxygen species (ROS); mitochondrial dysfunction; and neuroinflammation, all of which ultimately contribute to neurodegeneration. Drugs in parentheses are thought to inhibit the corresponding process. Modified from our previously published diagram [8].

Parkinson’s disease (PD) is the second most common neurodegenerative disorder affecting one million Americans and costing over USD 52 billion annually [1]. Despite extensive global research efforts, no neuroprotective or disease-modifying treatments for Parkinson’s disease (PD) have been established. However, it is well understood that several processes contribute to neurodegeneration in PD. These include: (a) the accumulation of misfolded alpha-synuclein, which is not cleared from the brain and triggers neuroimmune responses; (b) calcium excitotoxicity, which disrupts mitochondria and leads to energy depletion, contributing to neurodegeneration; (c) the build-up of iron in the substantia nigra, which activates microglia, causes neuroinflammation and generates reactive oxygen species (ROS); (d) mitochondrial dysfunction; and (e) neuroinflammation [2,3,4,5,6,7]. All of these processes contribute to and mediate neuronal damage, resulting in neurodegenerative conditions, as illustrated in Figure 1. Moreover, the progression of these processes varies among patients, occurring at different times and progressing at different rates for each individual. Therefore, testing a single medication that targets only one of these processes becomes challenging in a clinical trial with a diverse PD population. This is because not all participants may share the same level of dysfunction in the specific targeted mechanism at the same time. One potential solution is personalized medicine, where patients are identified based on the specific dysfunctional process affecting them, and targeted treatment is provided to address that issue. Alternatively, a more practical approach could involve using a “cocktail” of medications, with each drug designed to target one of the pathways contributing to neurodegeneration in PD. This combination therapy is likely more feasible and realistic, as it can be applied broadly to all PD patients.

There has been no rigorous comprehensive assessment of multiple drug combinations able to provide an additive disease-modifying effect for PD. Therefore, to conduct this drug-repurposing study, we selected nine existing medications to test in various combinations. These drugs have been previously assessed individually in both in vitro and in vivo models for PD, and some in human clinical trials. These include sodium phenylbutyrate, terazosin, exenatide, ambroxol, deferiprone, creatine, coenzyme Q10, dasatinib and tauroursodeoxycholic acid. These drugs were picked based on their diverse mechanisms of action, targeting various pathways involved in neurodegeneration, as illustrated in Figure 1. While they demonstrated strong efficacy in preclinical PD models, they either did not provide clinical evidence of disease modification or neuroprotection in trials or have yet to be investigated in PD patients.

## 2. Materials and Methods

### 2.1. Study Drugs

1. Sodium phenylbutyrate, an FDA-approved therapy for reducing plasma ammonia and glutamine in urea cycle disorders, acts as a chemical chaperone preventing misfolded α-synuclein aggregation [9]. Preclinical studies showed it halted disease progression in a chronic PD mouse model and may have therapeutic benefit in PD [10].

2. Terazosin, an FDA-approved therapy for hypertension and benign prostatic hyperplasia, enhances activity of phosphoglycerate kinase 1, thereby stimulating glycolysis and increasing cellular ATP. A retrospective study showed that people taking terazosin had a lower hazard ratio for developing PD [11].

3. Exenatide, an FDA-approved therapy for diabetes mellitus, is a glucagon-like-peptide-1 activator that may act as a microglia-deactivating factor [12]. It also can reduce accumulation of α-synuclein [13] and improve mitochondrial function [14,15]. We used exenatide natural analog exendin-4 in our experiments.

4. Ambroxol is a secretolytic agent used over-the-counter in other countries for the treatment of respiratory diseases associated with excessive mucus. It is reported to increase GCase activity in different brain regions, which may reduce accumulation of misfolded α-synuclein [16].

5. Deferiprone is an FDA-approved therapy for systemic iron overload that passes through the blood–brain barrier and chelates excessive brain iron from the substantia nigra. In a double-blind, placebo-controlled clinical trial in PD, it decreased substantia nigra iron deposits (on MRI) and improved motor scores of disease progression [17]. However, more recently, another double-blind, placebo-controlled study demonstrated worse symptomatic outcomes [18].

6. Creatine is a natural supplement and guanidine-derived compound that plays a key role in energy buffering within the cell [19]. Creatine protected cultured primary embryonal hippocampal and cortical cells against glutamate and calcium excitotoxicity in an in vitro model of neurodegeneration [20]. A multicenter, double-blind, parallel-group, placebo-controlled efficacy trial failed to show any clinical benefits [21].

7. Coenzyme Q10 (CoQ10) regulates ATP production and reduces free radical generation. CoQ10 also serves as an antioxidant in mitochondria. Additionally, CoQ10 inhibits glutamate excitotoxicity and oxidative-stress-mediated mitochondrial alteration in a mouse model of glaucoma [22]. Effects of CoQ10 in PD patients have been studied extensively and results are mixed. Interestingly, combined use of CoQ10 and creatine produced an additive neuroprotective effect against dopamine depletion in the striatum in mouse models of PD compared to either component alone [23].

8. Dasatinib is an FDA-approved c-Abl inhibitor for chronic myeloid leukemia. Another c-Abl inhibitor, nilotinib, reduced inflammation and enhanced autophagic clearance of α-synuclein in A53T transgenic mice [24]. However, in a placebo-controlled trial (NILO-PD), nilotinib did not meet outcomes of efficacy [25], likely due to poor CNS penetrance. In a recent study, dasatinib suppressed c-Abl activation in the mouse brain in the presence of 1-methyl-4-phenyl-1,2,3,6-tetrahydropyridine (MPTP) and appeared to be superior to nilotinib [26].

9. Tauroursodeoxycholic acid (TUDCA) is an endogenous bile acid that can act as a strong neuroprotective agent. TUDCA improves the survival of dopamine (DA) neurons in in vitro and in vivo experiments [27]. TUDCA attenuates mitochondrial dysfunction and ROS production in an MPTP mouse model of PD [28] and prevented autophagy, in addition to inhibiting alpha-synuclein (SYN) aggregation [29].

### 2.2. Model 1. Wild-Type Human Dopaminergic iPSC Line Treated with 1-Methyl-4-Phenylpyridinium (MPP+)

We have screened various combinations of the above medications for their neuroprotective effect against MPP+ toxin in human dopaminergic pluripotent stem cells (iPSCs) (Table 1). The selection of combinations for testing was based on utilizing different mechanisms of action of the individual drugs in each combination. Thus, each combination had drugs targeting at minimum: neuroinflammation, a-syn accumulation and mitochondrial dysfunction as shown in Figure 1. Deferiprone and terazosin were included as they can indirectly improve dopaminergic neurodegeneration. We initially tested 36 combinations of two to five drugs per group using iPSC-derived human dopaminergic neurons treated with MPP+. iCell DopaNeurons are neural floor-plate-derived midbrain dopaminergic neurons generated from human induced iPSCs. Dopaminergic neurons, specifically those located in the floor-plate-derived midbrain, are implicated in neurological disorders such as Parkinson’s disease, multiple system atrophy (MSA) and diffuse Lewy body disease (DLBD), among others. Thus, iCell DopaNeurons provide a highly relevant in vitro model to investigate these types of pathologies. The iCell DopaNeurons were supplied by FUJIFILM Cellular Dynamics, Madison, WI, USA.

Initially, we assessed the solubility of the drugs in the test media and ensured that neither the drugs nor the media had toxic effects on dopaminergic cells. We then compared the efficacy of those combinations in protecting against the toxic effect of MPP+ to that of the individual drugs and the control. The doses of the compounds were determined from existing literature and our previous testing trials as outlined below: PBA: 500 µM [10]; terazosin: 10 µM [30]; EXD: 100nM ([31] and Porsolt’s experience); ambroxol: 30 µM [32]; deferiprone: 50 µM [33]; creatine: 10 µM [34]; CoQ10: 1 µM [35]; and TUDCA: 50 µM [27]. Dasatinib was not tested in this experiment. The culture medium was BrainPhys™ Neuronal Medium, supplied by Stemcell Technologies, Vancouver, BC, Canada. Vehicles were: culture medium alone, 0.5% H_2_O and 0.16% DMSO in culture medium.

Cell culture: iCell DopaNeurons were thawed and cultured in BrainPhys medium with provided supplements, 1% N_2_ supplement (Stemcell Technologies), laminin and penicillin/streptomycin. They were plated at 20,000 cells per well in a 384 well plate (precoated with poly-D-lysine and laminin) in 70 μL of growth medium. Cells were maintained at 37 °C/5% CO_2_ in a humidified incubator for cell culture.

MPP+-induced neurotoxicity: Twenty-four hours after neuronal plating, half of the medium was removed, and the test compounds and MPP+ treatment (100 μM) were added to the wells. Three different measurements were made in the experiments: neurite length in mm, neurite branching points per mm^2^ and cytolysis of the cells as a percentage.

Neurite outgrowth was followed for 72 h using an Incucyte Zoom platform (Sartorius, Göttingen, Germany) with one phase-contrast image taken every four hours, using a 20× objective lens. After 72 h, the medium containing test compounds and MPP+ was removed and replaced with fresh medium containing a fluorescent cytolysis marker (red fluorescence). Each condition was tested in 8 wells of a 384-well plate. In this pivotal study we performed each experiment only once per combination tested. However, individual drugs were run multiple times on different plates with different combinations containing some of the individual drugs since all tested combinations could not fit on just one plate. In the MPP+ experiment eight wells were tested per condition in both experiments with iCell DopaNeurons.

#### Assay Endpoints and Image Analysis

Phase-contrast images were analyzed at each time point to determine the neurite length and number of branch points per mm^2^. Kinetic data were normalized by subtracting the value of the first data point (at time of treatment), providing a measure of changes in neurite outgrowth from the onset of the treatment, starting at zero. The area under the curve (AUC) of kinetic data was obtained and used for plotting compounds’ effects and performing statistical analysis. Fluorescent images were obtained at the endpoint to determine the number of dead cells and calculate the percentage of cytolyzed cells.

### 2.3. Model 2. α-Synuclein Triplication Human Cell Line

We evaluated effects of the four identified combinations from the previous experiment which showed the highest additive effects on human dopaminergic iPSCs treated with MPP+ using an α-synuclein triplication cell line (line 3x-1 [36], reprogrammed from a patient’s B-lymphocyte line GM15010 of the Coriell Cell Repository). This was performed at Northwestern University, Chicago, USA. Specifically, we studied sodium phenylbutyrate (PBA), tauroursodeoxycholic acid (TUDCA) and exendin (EXD) in different combinations. iPSC-derived dopamine (DA) neurons harboring triplication of the α-synuclein locus and an isogenic corrected line were plated at a density of ~1666 cells/mm^2^ on a 96-well glass bottom plate and cultured in SM1-supplemented neurobasal media for two months before beginning treatment with the four possible combinations of sodium phenylbutyrate (500 µM), tauroursodeoxycholic acid (50 µM) and exendin-4 (100 nM) for three months with twice-weekly media changes. We used 8 wells per vehicle control and 6 wells per testing condition. The samples were split over two plates, each of which included all individual drugs next to the combinations that they were a part of. Therefore, the individual drugs were run twice.

Following the treatment, cells were immediately fixed with 4% paraformaldehyde for 30 min at RT, washed three times with PBS, permeabilized with 0.2% Triton X-100 for 30 min, blocked with Odyssey Blocking Buffer for 60 min and incubated overnight at 4 °C with primary antibody against neurofilament (1:1000, Synaptic Systems 171 002). Cells were washed 3× with 0.1% Tween 20 in PBS and incubated with Alexa Fluor Goat Anti-Rabbit 790 (1:1000) and Goat Anti-Mouse 647 (1:1000) for 1 h at RT, before washing 3× with 0.1% Tween 20 in PBS and imaging with a Sapphire Biomolecular Imager (Azure biosystems, Dublin CA 94568, USA). Neurofilament heavy chain signal intensity was normalized to a fixed Hoechst signal measured with a Spectra Gemini plate reader (ex = 350 nm, em = 461 nM). For analysis, we excluded culture wells that had excessive cell clumping and therefore struggled to obtain an accurate quantification. One sample was excluded from the PBA alone group based on Grubb’s test for outliers using GraphPad Prism Version 10.3.1 (464).

### 2.4. Model 3. Human iPSC-Derived Idiopathic PD Cell Line

The use of human induced pluripotent stem cells (iPSCs) and iPSC-derived cells in this research was approved by the McGill University Research Ethics Board (IRB Study Number A03-M19-22A). Every person who contributed material for iPSC lines was at least 21 years old and provided a written informed consent. All experiments were performed in accordance with relevant guidelines and regulations and as per our recent report [8]. Specifically, the human iPSC lines used in this experiment included a sporadic PD line TD16 which was derived from the peripheral blood mononuclear cells (PBMCs) of a female patient diagnosed with sporadic PD. The age of collection for PBMCs was 61 years. The analysis of key genes associated with Parkinson’s disease, including SNCA, PARK2, PARK6, GBA1, LRRK2, TMEM175 and MAPT, did not show any mutations or variations, and no genetic factors were identified as the underlying cause for the patient’s condition. IPSCs were reprogrammed from PBMCs with episomal plasmids as described previously [37]. After obtaining iPSC clones, a thorough quality control process, as outlined previously, was conducted before they were deemed suitable for use. This quality control process involved assessing short tandem repeat (STR) profiles, testing for genome stability, conducting karyotyping, verifying pluripotency, checking for mycoplasma contamination and performing trilineage tests. The quality control procedure was outlined in a prior research investigation [37]. Dopaminergic neural progenitor cells (DA NPCs) derived from the sporadic PD line TD16 were systematically distributed into 96-well plates (15,000 cells per well) in dopamine differentiation medium and permitted to adhere for a period of 24 h. The mechanized process of cell seeding was accomplished using an EL-406 Washer Dispenser (BioTek, Winooski, VT, USA) for tasks such as plate coating, cell seeding, fixation and staining, as described [38]. Once seeded, the dopamine neurons were cultured in a maintenance solution, as detailed in previous reports [39,40]. Six duplicate plates were used for seeding each cell line. We used 6 replica plates with 2 wells per plate for a total of 12 wells per condition tested. The experiment was performed once. To avoid potential edge effects skewing the results, cells were only seeded into the inner 60 wells of each 96-well plate. The combinations tested are shown in Table 2.

### 2.5. Dasatinib-Containing Combination Testing in iPD TD16 Cell Line

The dose of dasatinib was 5 nM based on our prior dose-testing experiment. After a two-week period of differentiation, half of the medium was replaced with fresh medium and compounds and their combinations were introduced, including seven single compounds, eight combos and an untreated PD control. This was performed using an automated droplet dispenser (I.DOT, Dispendix, Stuttgart, Germany). Following a period of seven days, the process of replacing half of the medium and adding compounds/combos was repeated. Four days later, this procedure was once again carried out. After 4 more days, dopaminergic neurons were fixed for 15 min with 4% formaldehyde (ThermoFisher Scientific, 28908, Waltham, MA, USA) in PBS, permeabilized for 5 min with 0.2% Triton X-100 (Sigma-Aldrich, X-100, St. Louis, MO, USA) in PBS, then blocked for 1 h in 2% BSA and 0.02% Triton X-100 in PBS. Neurofilament Heavy (NFH) antibody (Sigma, cat# N4142) was diluted in blocking buffer (final dilution 1:2000) and added for overnight incubation at 4 °C. The cells were then washed three times with 0.2% Triton X-100 in PBS and a secondary antibody diluted in blocking buffer was added for one hour in the dark, then cells were washed three times with 0.2% Triton X-100 in PBS. Nuclei were stained with 2.5 μg/mL Hoechst33342 (ThermoFisher Scientific, H3570) in PBS for 10 min. After 2 additional washes with PBS, the cells were imaged on the Opera Phenix HCS system (Revvity, Waltham, MA, USA) with a 20× water immersion objective (NA 1.0) in confocal mode. Three z-positions were collected at a 2 μm interval to compensate fluctuations in the focal position of cells.

### 2.6. PBA/TUDCA/EXD Combo Testing in iPD TD16 Cell Line

We evaluated the effects of the 4 previously identified combinations from the previous experiment which showed additive effects on human dopaminergic iPSCs treated with MPP+ now using a sporadic PD human iPSC line. Specifically, we studied PBA, TUDCA and EXD in different combinations. After seven days of differentiation, half of the medium was replaced with fresh medium, and the compounds and their combinations (3 single compounds and 4 combos: PBA + EXD, PBA + TUDCA, TUDCA + EXD, PBA + TUDCA + EXD as well as untreated PD control) were added using a contact-free droplet dispenser (I.DOT, Dispendix). From this point on, exchange of half of the medium and the addition of compound/combinations were performed every 4 days. The incubation of the sporadic PD line TD16 was continued for a total of 4 weeks (total of 6 media/drug changes) before being fixed, stained and imaged as described in the previous section.

### 2.7. Image Analysis

The images were assessed using Harmony image analysis software (Revvity, V.5.1). Prior to analysis, the lighting in all images was adjusted and image stacks were merged using maximum projection. Nuclei stained with Hoechst33342 were recognized as objects in order to determine the number of cells per well. NFH staining was examined by setting thresholds on the images and filtering for cell bodies and filamentous structures (neurites). The identified objects were aggregated per image to compute the collective area taken up by neuronal cells in each image. The mean per well was then derived from the individual image data as the ultimate endpoint.

### 2.8. Model 4. Human Microglia Cell Line

We assessed the anti-neuroinflammatory properties of the 4 previously identified combinations from the previous experiment which showed additive effects on human dopaminergic iPSCs treated with MPP+. Specifically, we studied sodium phenylbutyrate (PBA), tauroursodeoxycholic acid (TUDCA) and exendin (EXD) in different combinations by assessing their impact on the secretion of pro-inflammatory cytokines in human brain microglia cells stimulated with lipopolysaccharide (LPS) and adenosine triphosphate (ATP). The doses selected were determined based on previously published studies: sodium phenylbutyrate = 500 µM [10]; exendin-4 = 100 nM [41]; TUDCA = 200 µM [42,43,44]. The combination of these 3 drugs was also evaluated for dose response at a 20% dose (100 µM, 20 nM and 40 µM, correspondingly). Microglia cells were obtained from an iCell^®^ Microglia kit, 01279 (Catalogue N° R1131) from FUJIFILM Cellular Dynamics. We adhered to the manufacturer’s guidelines to activate the microglial cells using LPS/ATP in order to assess the impact of the study drugs on the pro-inflammatory cytokines IL-6, TNF-α and CXCL1/GROα. The cell culture procedures were carried out under sterile conditions, within a laminar flow hood. The microglial cells were defrosted and nurtured in accordance with the instructions provided by the supplier. They were seeded at a density of 50,000 cells per well in a Corning Primaria 96-well plate (FisherScientific, Pittsburgh, PA, USA) using 100 μL of growth medium. The cells were then placed in a humidified cell culture incubator, set at 37 °C/5% CO_2_. Three days post-seeding, the test or reference compounds were administered 1 h prior to the stimulation with LPS and ATP. One hour later, the test or reference substances were added again followed by LPS. ATP was introduced at 5 h 30 min after incubation with LPS. After 6 h of exposure to LPS, the supernatant from each well was collected and centrifuged at a speed of 1000× *g* for 10 min to eliminate the cells and debris. Cytokine secretion (IL-6, TNF-α, CXCL1/GROα) was determined in the supernatant by the Luminex platform (BIO-RAD, Hercules, CA, USA) according to the provider’s instructions. Before conducting the assay, the samples were appropriately diluted to ensure they fell within the standard range. Each condition was tested using three wells. The levels of cytokines in the supernatant were quantified in pg/mL.

### 2.9. Statistical Analysis

For each experiment, the assumptions of normality were checked using a Shapiro–Wilk test and homoscedasticity analysis was performed using a Brown–Forsythe test before conducting ANOVA. When the assumptions failed (*p* < 0.05), log transformations and a Wilcoxon Mann–Whitney non-parametric rank-sum test were used to corroborate the results.

The analysis for a possible synergistic effect of a combination was a *t*-test comparison by calculating the difference between the combination effect and the sum of the individual drug effects after subtracting the control (vehicle) effect. This was run through an analysis of variance (ANOVA) using SAS V.9.4.

Further data processing was performed in Excel (Microsoft, Redmond, WA, USA) and Prism (GraphPad Software version 10.2.3). We normalized the outcomes of each treatment to the control group receiving only the vehicle. Statistical significance was assessed using one-way ANOVA and further confirmed with Bonferroni’s multiple comparison test. The results were shown by the mean values along with their standard deviations (s.d.).

## 3. Results

### 3.1. Model 1. Effect of Experimental Conditions on MPP+-Induced Toxicity in Human Dopaminergic iPSCs

**Figure 2:**
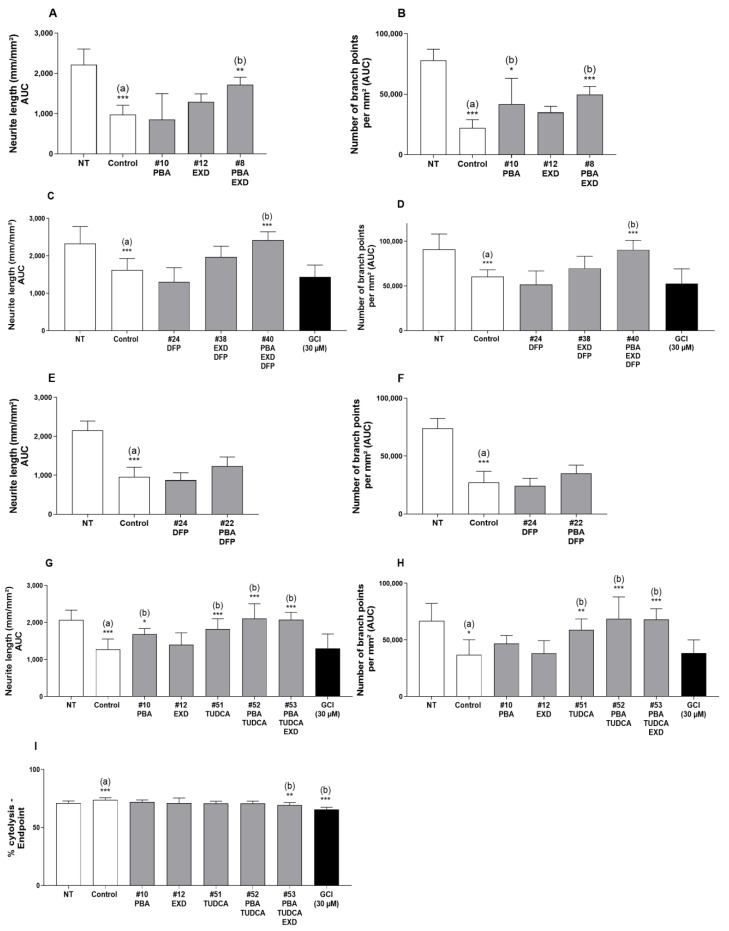
Effect of selected experimental conditions on Area Under Curve (AUC) of neurite length (mm/mm^2^) (**A**,**C**,**E**,**G**) and of number of branch points per mm^2^ (**B**,**D**,**F**,**H**), 72 h after MPP+ incubation in human dopaminergic iPSCs. The percentage of cytolysis was also determined by evaluating the number of dead cells as compared with the total number of cells at 72 h (**I**). (a): Compared with Non-Treated conditions (NT); (b): Compared with MPP+ controls (* *p* < 0.05; ** *p* < 0.01; *** *p* < 0.001).

**Figure 3:**
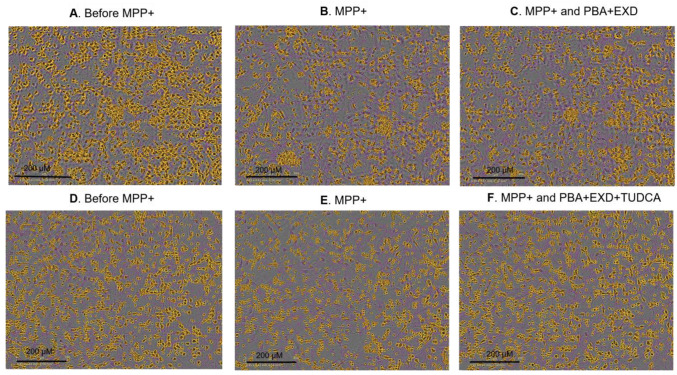
Neurite outgrowth and the number of branches 72 h after incubation with MPP+ and tested drug combinations. Artificial colors are applied on images to show the detection of neurites (pink) and cell bodies (orange) by Incucyte live-cell imaging platform (magnification ×20). (**A**,**D**) Non-treated conditions; (**B**,**E**) the same cells treated with MPP+ and (**C**,**F**) the same cells treated with MPP+ and corresponding drug combinations. PBA: Sodium phenylbutyrate; EXD: Exendin-4; TUDCA: Tauroursodeoxycholic acid.

After screening 36 drug combinations using the MPP+ human iPSC model and eight additional combinations using the iPD human iPSC line, we have identified four promising combinations. One combination (PBA and EXD, Figure 2A,B, sample #8) resulted in the neurite length and the number of branch points increasing by 75% and 125%, respectively, as compared with MPP+ alone (*p* ≤ 0.01 and *p* ≤ 0.001, respectively), Figure 3. When tested individually, these drugs (samples 10 and 12) showed a protective effect that was less than when the drugs were combined. The statistical test for synergism of this combination did not demonstrate sufficient significance with *p* = 0.0644. We identified another combination of PBA, EXD and DFP which showed an additive protective effect (Figure 2C,D, sample #40). The neurite length and the number of branches were increased by 48.9% and 49.7%, respectively, as compared with MPP+ alone (*p* ≤ 0.001 for both). These drugs tested individually or in combinations PBA + DFP and EXD + DFP (Figure 2C–F), however, did not show a statistically significant protective effect. We also found that combinations of TUDCA and PBA (Figure 2G–I, sample 52), as well as TUDCA, PBA and exendin-4 (sample 53), increased the neurite length (65.2% and 63.2%) and the number of branches (86.6% and 85.1%, respectively) as compared with control/MPP+-treated cells without the drugs (Figure 2G–I, *p* ≤ 0.001 for both conditions). The latter combination (#53) also reduced cytolysis of the cells which was statistically significant and was not seen with any other combinations (Figure 2I and Figure 3). While TUDCA alone (sample 51) protected against MPP+ toxicity as well, the effect was at least 27% greater when the drugs were combined. Other tested combinations did not produce any statistically significant additive effects (Figure S1).

### 3.2. Model 2. Effect of Experimental Conditions on α-Synuclein Triplication Cell Line

**Figure 4:**
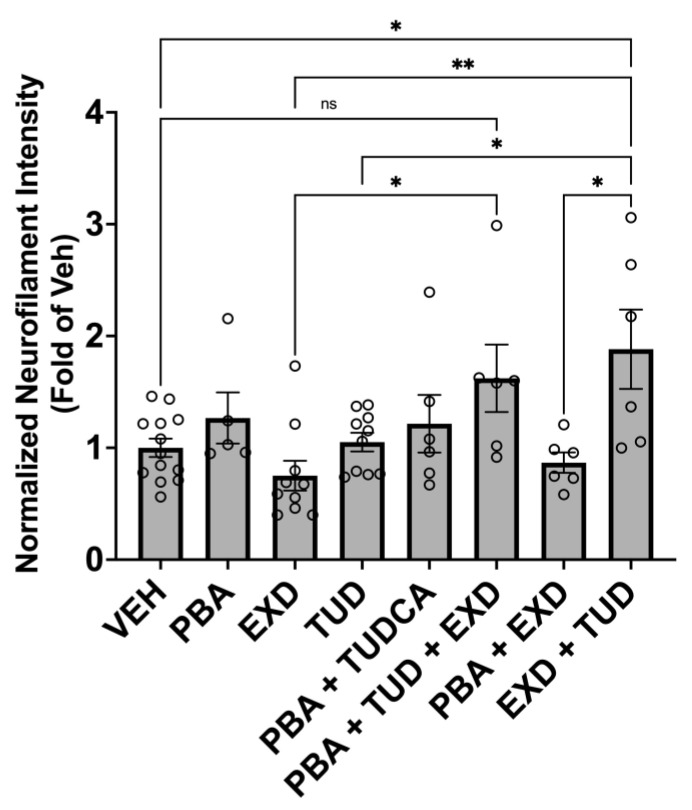
Effect of experimental conditions on Neurofilament Heavy Chain signal intensity in alpha-synuclein triplication cell line. Line 3x-1 at day 60 postdifferentiation was treated with the indicated compounds for 3 months, then fixed and immunostained for neurofilament protein as an indication of neurite health. Values are the mean +/− SEM, ANOVA with Tukey’s post hoc test (* *p* < 0.05; ** *p* < 0.01; ns—not significant). Each plot represents an individual culture well. EXD, Exendin-4; TUD, TUDCA; PBA, sodium phenylbutyrate; VEH, vehicle.

The α-synuclein triplication cell line was matured to day 60, then treated with the combinations of: TUDCA/exendin, TUDCA/PBA, TUDCA/exendin/PBA, exendin/PBA for 90 days. This showed a 52% increase in neurofilament heavy chain signal intensity with the TUDCA/exendin combo compared to the cells that were not treated (*p* < 0.01) and the two drugs individually (*p* < 0.01 and <0.001). While none of the other possible combinations of PBA, TUDCA and exendin showed any statistically significant changes over the untreated cells of this line, the triple combination PBA/TUDCA/EXD showed a positive trend with *p* = 0.16 (Figure 4).

### 3.3. Model 3. Effect of Experimental Conditions on TD16 Idiopathic PD Cell Lines

**Figure 5:**
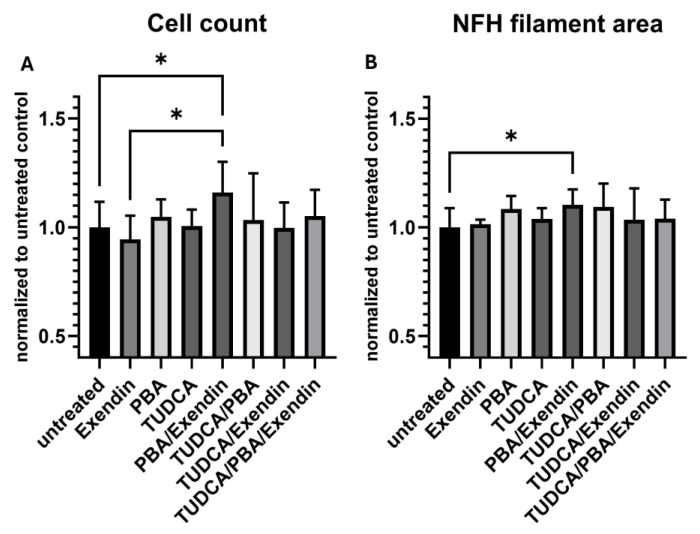
Effects of compound treatment on cellular state of idiopathic PD cell line. (**A**) Cell count: the cell (nuclei) count is based on the number of Hoechst-stained nuclei per well. Each bar represents the normalized mean cell count per well. (**B**) Neurite network density: neurites are stained with an antibody against Neurofilament heavy chain, marking all neurites. Each bar represents the mean NFH-positive area per well. For (**A**,**B**), the per well values are normalized against the mean of all untreated wells. Error bars represent standard deviation. (* *p* < 0.05).

The TD16 Parkinson’s dopaminergic cell line which was treated with the combination of PBA/exendin for 28 days showed a 16% increase in cell count compared to the untreated cells (*p* < 0.01). The NFH filament area exhibited a 10% increase (significant at *p* < 0.05) in comparison to the cells that were not treated (Figure 5). Neither EXD nor PBA alone showed an increase in the cell count or NFH filament area though.

None of the eight selected combinations containing dasatinib showed any additive or synergistic effects on cell count or NFH (Figure S1).

### 3.4. Model 4. Effect of Experimental Conditions on Human Microglia Cell Line

**Figure 6:**
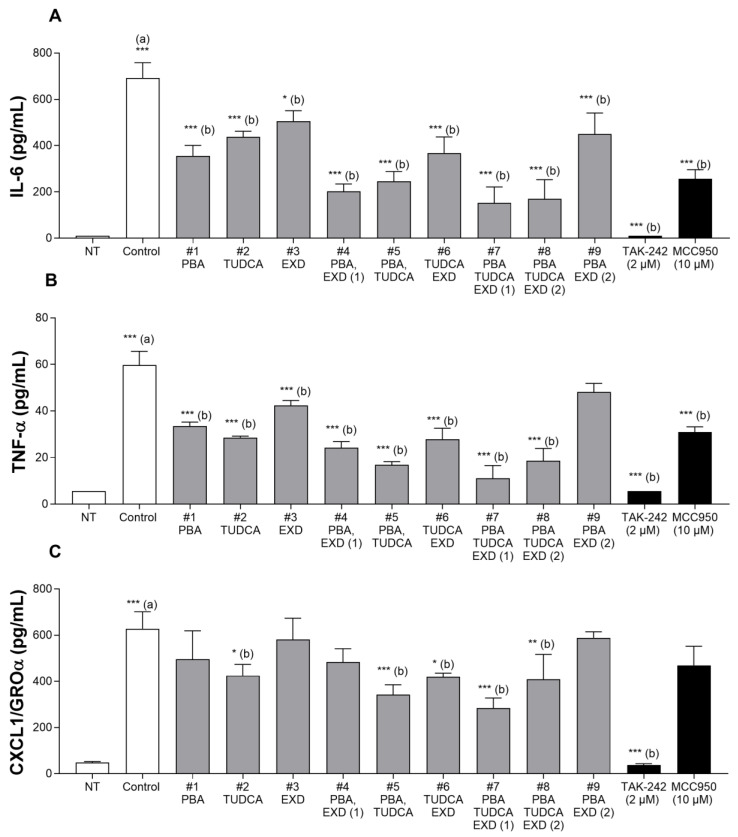
Effect of experimental conditions on pro-inflammatory cytokines in human Microglial cells 6 h after LPS incubation (ATP was added 5.5 h after LPS). (**A**). Effect on IL-6; (**B**). Effect on TNF-α; (**C**). Effect on CXCL1/GROα. 1. PBA: Sodium phenylbutyrate at 500 µM; 2. TUDCA at 200 µM; 3. EXD: Exendin at 100 nM; 4. PBA at 500 µM and EXD at 100 nM; 5. PBA at 500 µM and TUDCA at 200 µM; 6. TUDCA at 200 µM and EXD at 100 nM; 7. PBA at 500 µM, TUDCA at 200 µM and EXD at 100 nM; 8. Lower doses of PBA at 100 µM, TUDCA at 40 µM and EXD at 20 nM; 9. Lower doses of PBA at 100 µM and EXD at 20 µM. (a): Compared with Non-Treated conditions (NT); (b): compared with LPS/ATP controls (* *p* < 0.05; ** *p* < 0.01; *** *p* < 0.001).

In the presence of LPS and ATP, the concentrations of IL-6, TNF-α and CXCL1/GROα were significantly increased, as compared with non-treated conditions (Figure 6A–C, correspondingly).

Condition 1: PBA significantly decreased the levels of IL-6 by 49% (*p* ˂ 0.001) and TNF-α by 44% (*p* ˂ 0.001), as compared with 0.58% for distilled water. Condition 2: TUDCA significantly decreased the levels of IL-6 by 37% (*p* ˂ 0.001), TNF-α by 52% (*p* ˂ 0.001) and, to a lesser extent, GROα by 32% (*p* ˂ 0.05) as compared with distilled water. Condition 3: EXD decreased the levels of IL-6 by 27% (*p* ˂ 0.05) and TNF-α by 29% (*p* ˂ 0.001), as compared with distilled water. Condition 4: The combination of PBA and EXD significantly decreased the levels of IL-6 (by 60%) and TNF-α (by 43%), as compared with EXD alone (both *p* ˂ 0.001). Condition 5: The combination of PBA and TUDCA significantly decreased the levels of IL-6 by 44%, as compared with TUDCA alone and the levels of TNF-α by 50%, as compared with PBA alone (*p* ˂ 0.01 for each cytokine). Condition 6: The combination of TUDCA and EXD significantly decreased the level of TNF-α by 34%, as compared with EXD alone (*p* ˂ 0.01). Condition 7: The combination of PBA, TUDCA and EXD resulted in a substantial reduction of IL-6 by 57% and TNF-α by 67% as compared with PBA alone (*p* ˂ 0.01 and *p* ˂ 0.001, respectively), 65% and 61% vs. TUDCA alone (*p* ˂ 0.001 for both cytokines) and 70% and 74% vs. EXD alone (*p* ˂ 0.001 for both cytokines). It also significantly decreased the level of GROα, as compared with PBA alone by 43% (*p* ˂ 0.05) and as compared with EXD alone by 51% (*p* ˂ 0.001). Condition 8: Lower doses of PBA, TUDCA and EXD (#8) decreased the levels of IL-6 (*p* ˂ 0.001), TNF-α (*p* ˂ 0.001) and GROα (*p* ˂ 0.01), as compared with distilled water. Condition 9: Lower doses of PBA and EXD (#9) decreased the level of IL-6 (*p* ˂ 0.001), as compared with distilled water (Figure 6A–C).

## 4. Discussion

We have identified four combinations of the three drugs described above for further validation. Indeed, all the tested medications in different combinations are thought to affect most of the recognized pathways such as aggregation of misfolded proteins, neuroinflammation, mitochondrial dysfunction, ROS formation and iron accumulation leading to neurodegeneration in PD (Figure 1). All four combinations of PBA + EXD, PBA + TUDCA, EXD + TUDCA and PBA + TUDCA + EXD improved neurite length, branching points of dopaminergic neurons or NFH. Interestingly, the combination of PBA and TUDCA is approved for ALS but was recently discontinued due to lack of efficacy in a Phase III clinical trial. In our study, this combination showed efficacy against MPP+-induced toxicity in the human dopaminergic iPSC model we used but did not reduce cytolysis. Addition of exendin-4 to that combination, however, did produce modest, but statistically significant, improvement in cell death (combination 53) and improved anti-inflammatory effects as demonstrated by reduction of pro-inflammatory cytokines. We believe that the addition of EXD to PBA and/or TUDCA provides significant benefits such as reduction in microglial activation and insulin resistance as well as further improvement of mitochondrial dysfunction, making this the most promising drug combination we have identified so far. The recent positive Phase II clinical trial of another GLP-1 agonist, lixisenatide, in PD points out the usefulness of this class of drugs in PD [45]. Exenatide and lixisenatide are the only approved GLP-1 agonists that cross the blood–brain barrier sufficiently [46]. However, this triple combination of PBA + TUDCA + EXD did not show a similar effect in the iPD iPSC line. Combination #40 containing deferiprone is also interesting, however, in the light of the recent negative large clinical study of deferiprone in drug-naïve PD patients which showed symptomatic worsening in the active arm [18], this drug should not be pursued at this time.

Anti-inflammatory effects of the individual drugs were observed with PBA at 500 µM, TUDCA at 200 µM and EXD at 100 nM. These effects were potentiated when the treatments were applied in different combinations. The most impressive results were seen with a triple combination of PBA at 500 µM, TUDCA at 200 µM and EXD at 20 nM (#7), which was superior to all single and all double combinations and statistically significantly decreased levels of all three cytokines studied, reducing the activation of the microglial cells (Figure 6A–C). A combination of PBA and EXD also produced a significant combinatory effect compared to the individual drugs. The effects were also observed with the combination of the three treatments at lower doses, i.e., PBA at 100 µM, TUDCA at 40 µM and EXD at 20 nM, but they were slightly lower, suggesting a dose–response effect. Because of the significance of neuroinflammation in dopaminergic neurodegeneration (Figure 1), these results demonstrate promise in the treatment of all synucleinopathic diseases.

This study has several limitations. We were unable to show the same universal effects of one combination in all in vitro models we used. This could be explained by the diversity of PD pathology and limitation of the disease models used. Besides the initial screening using an MPP+-treated wild-type human dopaminergic cell line and alpha-synuclein triplication line, we assessed a single cell line obtained from a patient with idiopathic Parkinson’s disease. Additional validation of the data is required using cell lines from patients with both sporadic and hereditary forms of PD. However, each of the three promising combinations identified separately using different cell lines contained exendin, suggesting it is an essential component to show additive effects. This is especially interesting in light of the recent clinical trial finding another GLP-1 agonist, lixisenatide (a close homolog of exenatide), showed slower progression of motor disability in early PD [45]. Potentially, a GLP-1 agonist could be combined with other classes of neuroprotective drugs currently in development.

The microglia experiment was conducted using wild-type human microglial cells activated with LPS, rather than cells derived from actual PD patients. Although this model is suitable for generalizing results across NDs, it would be significant to validate the anti-inflammatory potential of the four combinations on microglial cells obtained from patients with an ND. Eight dasatinib-containing combinations were not tested in the initial screening process using the MPP+-treated iPSC line or in the alpha-synuclein triplication line. While we tested different doses of the individual drugs prior to the screening of combinations, we did not test multiple doses of a given combination except for in the microglia experiment. However, to overcome this, we evaluated relative additive effects of the combinations compared to their individual components. We were not able to prove the synergistic effect of any combination, but the PBA + EXD combo showed a positive trend for synergism with *p* = 0.0644 in the MPP+-treated wild-type human dopaminergic iPSC line. This is probably due to the small number of samples tested. We did not test all possible combinations of the nine selected drugs, but only those that could affect at least three main processes leading to neurodegeneration: mitochondrial dysfunction, alpha-synuclein accumulation and neuroinflammation, based on the published literature. It is possible some other drug combinations not tested possess these properties too.

Overall, this work represents the first detailed in vitro evaluation and conceptual development of drug combinations that target multiple mechanisms. This could lead to the development of a drug candidate capable of neuroprotection and offering clinically significant outcomes in idiopathic PD. A larger study employing additional multiple iPD patient-derived cell lines including microglia should be conducted to reconfirm our findings and identify one combination showing neuroprotection in the majority of cell lines from sporadic cases of PD, making it applicable to a larger patient population.

## Figures and Tables

**Table 1:** Combinations tested in WT human dopaminergic iPSC line treated with MPP+.

#	Drugs in Combination	#	Drugs in Combination
1	PBA, TZ, EXD, CR, CoQ10, Medium	23	AMB, MPP+
2	PBA, TZ, EXD, CR, CoQ10, MPP+	24	DFP, MPP+
3	TZ, EXD, CR, CoQ10, MPP+	25	PBA, TZ, EXD, AMB, DFP, Medium
4	EXD, CR, CoQ10, MPP+	26	PBA, TZ, EXD, AMB, DFP, MPP+
5	PBA, TZ, CR, CoQ10, MPP+	27	PBA, TZ, AMB, DFP, MPP+
6	PBA, TZ, EXD, MPP+	28	PBA, TZ, DFP, MPP+
7	TZ, EXD, MPP+	29	TZ, EXD, AMB, DFP, MPP+
8	PBA, EXD, MPP+	30	EXD, AMB, DFP, MPP+
9	PBA, TZ, MPP+	31	PBA, EXD, AMB, DFP, MPP+
10	PBA, MPP+	32	PBA, TZ, EXD, DFP, MPP+
11	TZ, MPP+	33	TZ, AMB, DFP, MPP+
12	EXD, MPP+	34	TZ, EXD, AMB, MPP+
13	CR, CoQ10, MPP+	35	TZ, DFP, MPP+
14	PBA, CR, CoQ10, MPP+	36	TZ, AMB, MPP+
15	TZ, CR, CoQ10, MPP+	37	EXD, AMB, MPP+
16	EXD, CR, CoQ10, MPP+	38	EXD, DFP, MPP+
17	PBA, TZ, EXD, AMB, MPP+	39	TZ, EXD, DFP, MPP+
18	PBA, TZ, AMB, MPP+	40	PBA, EXD, DFP, MPP+
19	AMB, DFP, MPP+	41	PBA, EXD, AMB, MPP+
20	PBA, AMB, MPP+	51	TUDCA, MPP+
21	PBA, AMB, DFP, MPP+	52	PBA, TUDCA, MPP+
22	PBA, DFP, MPP+	53	PBA, TUDCA, EXD, MPP+

Codes: PBA—Sodium phenylbutyrate; TZ—Terazosin; EXD—Exendin-4; AMB—Ambroxol; DFP—Deferiprone; CR—Creatine; CoQ10—Co-enzyme Q10; TUDCA—Tauroursodeoxycholic acid; entries 42–50 were skipped.

**Table 2:** Combinations tested in human iPD dopaminergic iPSC line.

Drugs in Combination	Drugs in Combination
Dasatinib, TUDCA	Dasatinib, Creatine, CoQ10
Dasatinib, EXD	Dasatinib, Terazosin
Dasatinib, PBA	PBA, EXD
Dasatinib, TUDCA, PBA	PBA, TUDCA
Dasatinib, PBA, EXD	TUDCA, EXD
Dasatinib, TUDCA, EXD	PBA, TUDCA, EXD

Codes: PBA—Sodium phenylbutyrate; EXD—Exendin-4; TUDCA—Tauroursodeoxycholic acid.

## Data Availability

The data that support the findings of this study are available from the corresponding author upon reasonable request.

## Supplementary Materials

The following supporting information can be downloaded at: https://www.mdpi.com/article/10.3390/antiox14040396/s1, Figure S1: A, B, C: Effect of selected experimental conditions on Area Under Curve (AUC) of neurite length (mm/mm^2^) and of number of branch points per mm^2^, 72 h after MPP+ incubation in human dopaminergic iPSC cells. (a) compared with Non-Treated vehicle (NT/NT) (b): compared with Non-Treated controls (control/NT); (c): compared with MPP-treated controls (control/MPP+) (* *p* < 0.05; ** *p* < 0.01; *** *p* < 0.001). D: Effects of Dasatinib-containing combination treatments on cellular state of idiopathic PD cell line: Cell count: the cell (nuclei) count is based on the number of Hoechst stained nuclei per well. Each bar represents the normalized mean cell count per well; Neurite network density: neurites are stained with an antibody against Neurofilament heavy chain, marking all neurites. Each bar represents the mean NFH-positive area per well. Values are normalized against the mean of all untreated wells. Error bars represent standard deviation. Supplemental Table S1: Materials used.
