# Supplementary material for: Evaluation of Additive Neuroprotective Effect of Combination Therapy for Parkinson’s Disease Using In Vitro Models
Source: Antioxidants (Basel). 2025 Mar 27;14(4):396. doi: 10.3390/antiox14040396 (PMC12024093; doi:10.3390/antiox14040396)
Supplement: Supplementary file 1 [file antioxidants-14-00396-s001.zip › Supplementary Table S1.pdf]

Supplemental Table S1: Materials used

Study Drugs:

**Compound**	**Abbreviation**	**Company**	**Cat#**	**Final concentration**
Tauroursodeoxycholic acid	TUDCA	Focus Biomolecules	10-2782	50uM
Sodium Phenylbutyrate	PBA	AdooQ	A16714	500uM
Exendin	EXD	Sigma	E7144	100nM
Dasatinib	DAS	Cayman Chem.	11498	5nM
Creatine	CR	Sigma	C0780	10µM
Coenzyme Q10	CoQ10	TOCRIS	3003	1µM
Terazosin	TZ	TOCRIS	1506	10µM
Ambroxol	AMB	TOCRIS	2404	30µM
Deferipone	DFP	Sigma Aldrich	Y0001976	50µM

**Materials for iPSC midbrain neurons and microglia**		
Anti-neurofilament antibody	Synaptic Systems	Cat# 171 002, RRID:AB_887743
Secondary antibody: Alexa Fluor 647 Goat anti-mouse IgG secondary (H+L)	Thermo Fisher Scientific	Cat #A21236; RRID: AB_144696
L-glutamine	Gibco	Cat #25030081
Glutaraldehyde, 25% aqueous solution	Electron Microscopy Sciences	Cat #16220
Penicillin / Streptomycin	Thermo Fisher Scientific	Cat #10378016
Matrigel	Fisher	Cat #CB-40234
mTeSR1 media	StemCell Technologies	Cat #85850
Neurobasal SM1 media	Thermo Fisher Scientific	Cat #21103-049
NeuroCult SM1 supplement	StemCell Technologies	Cat #05711
Vitronectin	StemCell Technologies	Catalog # 07004
6-well plates for culturing iPSCs	Corning	Corning Catalog #354277
Knockout DMEM	Thermo Fisher Scientific	10829-018
Y-27632	Sigma / Millipore	688000-1MG

SB 431542, 1 mg	R & D systems	1614/1
RecHu BDNF, 5ug	R & D systems	11166-BD
ReHu SONIC Hedgehog	R & D systems	1845-SH-025
RecHu FGF-8a isoform	R & D systems	4745-F8-050
RecHu GDNF	R & D systems	212-GD-010
RecHuTGF-beta 3	R & D systems	243-B3-002
dibutyryl cAMP	Enzo	BML-CN125-0100
Laminin (from mouse EHS sarcoma)	Sigma / Roche	11243217001
poly D- Lysine	Sigma	P1149-10MG
iCellDopa kit, 01279	FUJIFILM Cellular Dynamics	R1088
MPP+	Sigma	D048
YOYO-3 fluorescent cytolysis marker	Fisher Scientific	Y3606
SYTO™ Green Fluorescent Nucleic Acid Stains	Fisher Scientific	S7559
iCell Microglia kit, 01279	FUJIFILM Cellular Dynamics	R1131
Lipopolysaccharide	Invivogen	tlrl-eblps
Adenosine 5'-triphosphate disodium salt	Invivogen	Tlrl-atpl
TAK-242 (Resartovid**)**	Invivogen	S7455
MCC950	Invivogen	Inh-mcc
**Materials for iPD TD16 cell line**		
Matrigel	Millipore	354277
Gentle cell dissociation reagent	STEMCeLL Technologies	07174
DMEM/F12	Thermo Fisher Scientific	10565042
N2	Thermo Fisher Scientific	17502048
B27	Thermo Fisher Scientific	17504044
NEAA	Thermo Fisher Scientific	11140050
BSA	Thermo Fisher Scientific	15260037
Noggin	Peprotech	120-10C
SHH	GenScript	Z03067
CHIR-99021	Selleckchem	S7146
SB431542	Selleckchem	S1067
FGF-8	PeproTech	100-25
Fetal bovine serum	Thermo Fisher Scientific	12484028

DMSO	Thermo Fisher Scientific	BP231-1
Poly-L-Ornithin	Sigma	P3655
Laminin	Sigma	L2020
STEMdiff Neural Progenitor Basal Medium	STEMCELL Technologies	05834
STEMdiff Neural Progenitor Supplement A	STEMCELL Technologies	05836
STEMdiff Neural Progenitor Supplement B	STEMCELL Technologies	05837
Purmorphamine	Sigma	SML-0868
StemPro Accutase dissociation reagent	Thermo Fisher Scientific	A1110501
BrainPhys Neuronal medium	STEMCELL Technologies	05790
N2A Supplement A	STEMCELL Technologies	07152
Neurocult SM1 Neuronal Supplement	STEMCELL Technologies	05711
BDNF	PeproTech	450-02
GDNF	PeproTech	450-10
Compound E	STEMCELL Technologies	73954
Db-cAMP	Biosynth	ND07996
Ascorbic acid	Sigma	A5960
96 well microplates	Corning, Falcon	353219
16% Formaldehyde	Thermo Fisher Scientific	28908
Hoechst33342	Thermo Fisher Scientific	H3570
Anti-neurofilament 200 antibody	Sigma	N4142 RRID:AB_477272
Donkey anti-rabbit IgG Alexa 546	Thermo Fisher Scientific	A10040 *RRID:AB_2534016*
