# Supplementary figures and images for: Evaluation of Additive Neuroprotective Effect of Combination Therapy for Parkinson’s Disease Using In Vitro Models
Source: Antioxidants (Basel). 2025 Mar 27;14(4):396. doi: 10.3390/antiox14040396 (PMC12024093; doi:10.3390/antiox14040396)

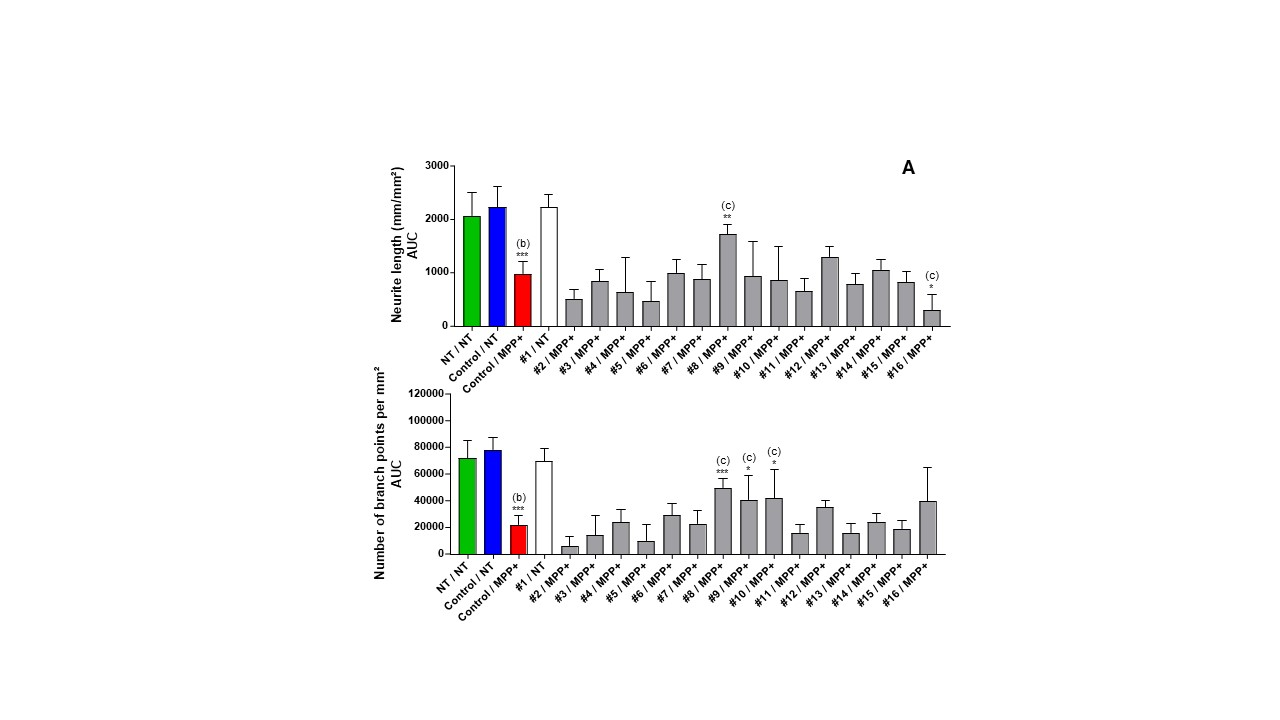

Supplement: Supplementary file 1 [file antioxidants-14-00396-s001.zip › Suppl Figure S1A.tiff]

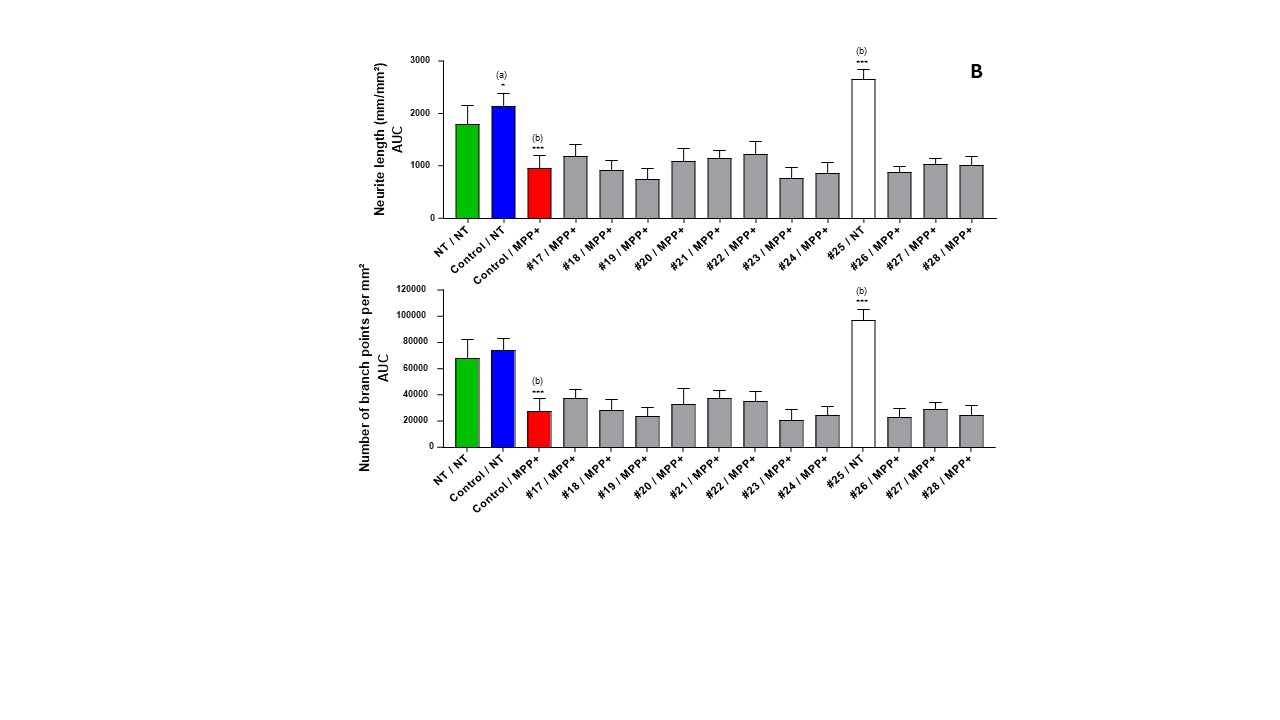

Supplement: Supplementary file 1 [file antioxidants-14-00396-s001.zip › Suppl Figure S1B.tiff]

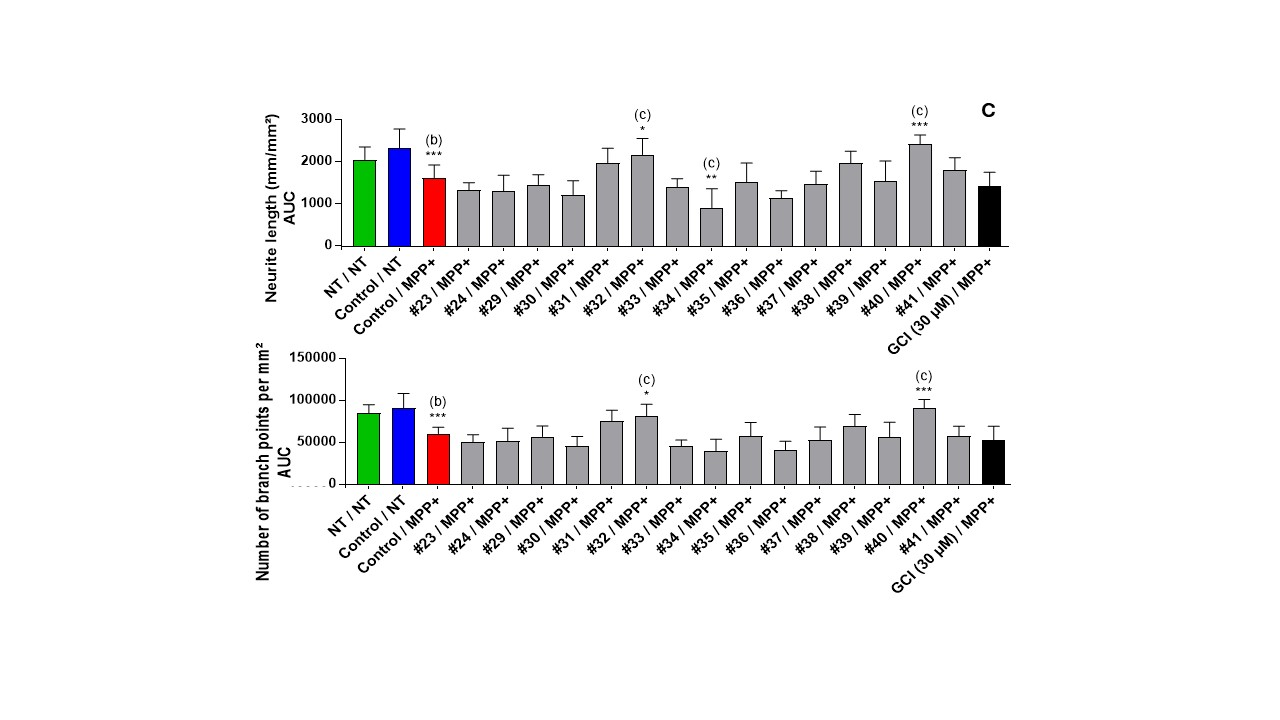

Supplement: Supplementary file 1 [file antioxidants-14-00396-s001.zip › Suppl Figure S1C.tiff]

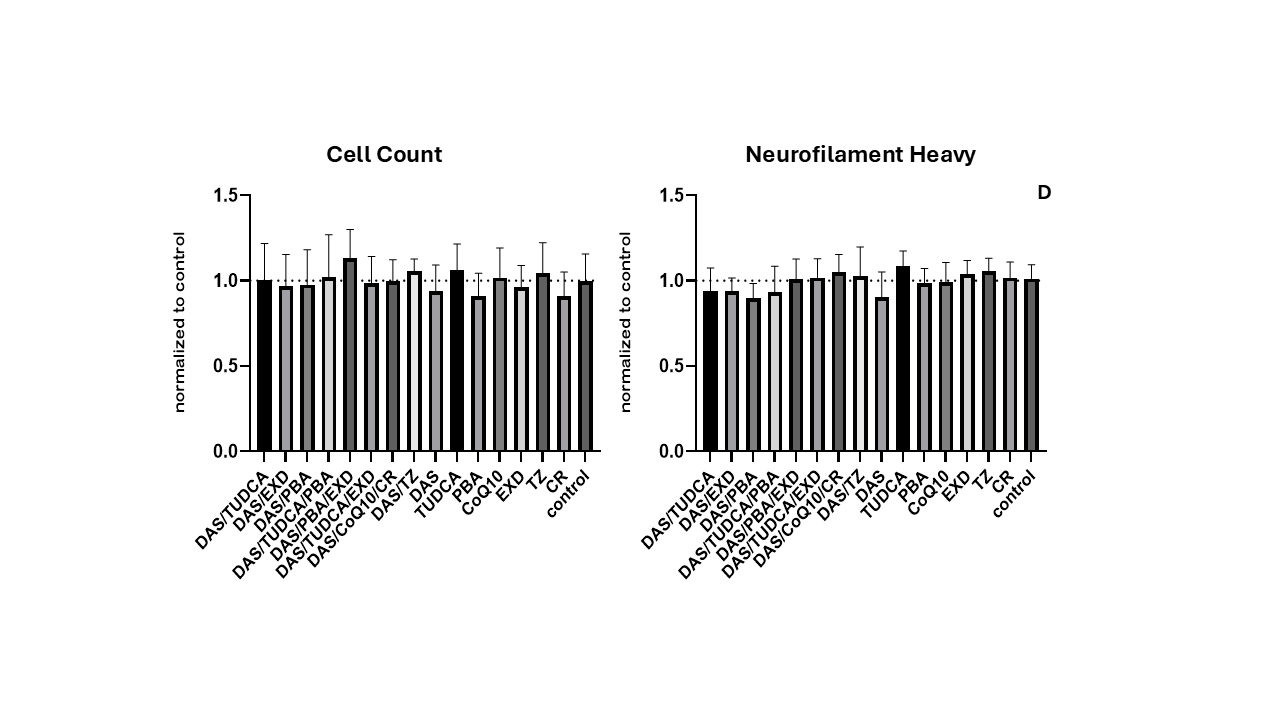

Supplement: Supplementary file 1 [file antioxidants-14-00396-s001.zip › Suppl Figure S1D.TIF]
